# The Development of Quality Indicators to Assess Family Wellbeing Outcomes Following Engagement with Children’s Mental Health Services in Ontario, Canada

**DOI:** 10.3390/ejihpe15100212

**Published:** 2025-10-15

**Authors:** Shannon L. Stewart, Boden D. Brock, Abigail Withers, Renee M. Guerville, John N. Morris, Jeffrey W. Poss

**Affiliations:** 1 Faculty of Education, University of Western Ontario, London, ON N6A 3K7, Canada; 2 Hebrew Senior Life Marcus Institute for Aging Research, Boston, MA 02131, USA; 3 School of Public Health Sciences, University of Waterloo, Waterloo, ON N2L 3G1, Canada

**Keywords:** interRAI, quality indicators, family functioning, parenting strengths, caregiver distress

## Abstract

(1) Background: Caregivers and families of children involved with mental health services face unique challenges. In Ontario, there is a dearth of information on outcomes for families following a child’s involvement with mental health services. Metrics known as Quality Indicators (QIs) offer a way to better understand these outcomes. Importantly, QIs can be risk adjusted to account for the influence of client complexity to allow for fair inter-agency comparisons. This study developed a set of risk-adjusted caregiver/family outcome QIs for children’s mental healthcare agencies. (2) Methods: Archival data from widely implemented interRAI child and youth assessment instruments was used. Previous methodology for QI calculation and risk adjustment was adapted and tested. (3) Results: Utilizing the interRAI suite of child and youth assessment instruments, a set of six QIs focusing on improvement or decline in parenting strengths, caregiver distress, and family functioning were developed. (4) Conclusions: The QIs established were sufficiently independent to represent different aspects of family wellbeing while the risk adjustment strategy developed was useful in removing client complexity from QI calculation. Implications for future directions, including the use of QIs at a systems level to more accurately direct resources and set performance benchmarks, are discussed.

## 1. Introduction

It is imperative that children’s mental health stakeholders view psychological development and wellbeing within the context of broader ecological influences, such as the family system and parental wellbeing (Bronfenbrenner, 1986; Gaspar et al., 2022; Kiani et al., 2016). The family–child influence can be described as transactional in nature, where the parent or caregiver (hereafter referred to as parent) and child impact each other simultaneously (Sameroff, 2009). The ability to measure, track, and compare the influence of agency-delivered intervention on family functioning, parenting, and caregiver distress is valuable in understanding the quality of care received.

### 1.1. Family Functioning and Parenting

Family functioning can be described as the abilities, roles, challenges, and repetitive behaviour of individuals within a family system (Gaspar et al., 2022; Kiani et al., 2016). Positive or strong family functioning has been associated with children’s wellbeing, conflict resolution skills, socio-emotional skills, and happiness (Gaspar et al., 2022; Hooper et al., 2023; Izzo et al., 2022; Kiani et al., 2016). Parenting, a key component of family functioning, is the act of socializing a child based on goals and values held by the parent (Darling & Steinberg, 1993). The extant literature posits that authoritative parenting, the appropriate balancing of warmth and control, contributes to positive child development (Baumrind, 2013; Pinquart, 2017a, 2017b). In a meta-analytic review examining child psychological adjustment and parental warmth, child hostility and aggression were negatively correlated with parental warmth and affection (Khaleque, 2013). Parental warmth was also related to positive self-esteem, emotional stability, emotional responsiveness, and other positive traits such as child independence and positive outlook (Khaleque, 2013). Examples of beneficial appropriate control in parenting practices include appropriately supervising the child, setting limits, and utilizing appropriate disciplinary practices (Almas et al., 2011; Sege et al., 2018; Oltean et al., 2020). Further, a parent’s sensitivity to their child’s needs has also been demonstrated to impact a child’s behavioural wellbeing, particularly with regard to externalizing difficulties (e.g., aggression; Cooke et al., 2022).

Family communication is another aspect of family functioning that can impact a child’s emotion regulation, mental health, and social wellbeing (Grey et al., 2022; Stewart et al., 2021b). For instance, although normative parent–child communication fluctuates over time, especially in adolescence, research suggests that quality parent–child communication is associated with positive academic outcomes, reducing health risk behaviours (e.g., alcohol and drug use), and with favourable mental health outcomes (Keijsers & Poulin, 2013; Riesch et al., 2006; Zapf et al., 2024; Zhang, 2020). Regarding mental health outcomes, in a recent systematic review, there were small-to-moderate effects of parent–adolescent communication on mental health overall, yet in cases of depression, post-traumatic stress disorder (PTSD), and psychosis, communication became more important to prioritize (Zapf et al., 2024). With regard to PTSD, communication has been found to be particularly important after a child’s traumatic experience as sensitive and responsive parent–child communication has been found to contribute to positive adjustment and psychological wellbeing following trauma (Sloover et al., 2024). These findings are of importance given that over half of the referrals to children’s mental health agencies have experienced some type of trauma and that potentially traumatic experiences for children can also result in mental health challenges for parents and family members (Wilcoxon et al., 2021).

### 1.2. Distress of Parents

Parental distress can negatively impact child-rearing and family functioning, often resulting in increased control and high-powered discipline characteristic of authoritarian parenting (Crnic & Ross, 2017; Fang et al., 2024; Fonseca et al., 2020; Hadjicharalambous & Demetriou, 2021). Unsupportive environments, fostered through harsh, critical, and high-powered parenting practices, have been linked to mental health and emotion dysregulation in children (Berla et al., 2022). Notably, harsh disciplinary practices have been related to child behaviour difficulties and poor academic performance and can impact brain development, all of which may contribute to cognitive and neurodevelopmental pathways to mental illness in children (Whittle et al., 2022, 2025; Wiggers & Paas, 2022). In addition, children of parents who are highly sensitive to external influences (to the point of often being overwhelmed) demonstrate a potential desensitization to negative social interactions, perhaps due to their parents’ use of inappropriate parenting practices in the face of distress (Christou et al., 2025).

Parental distress is influenced by parent, child, and situational factors and can contribute to poor child outcomes (Abidin, 1992; Fang et al., 2024). Specifically, children of highly stressed parents are at heightened risk for internalizing and externalizing psychological symptoms, conduct difficulties, hyperactivity, and increased levels of overall psychological distress into adulthood (Amrock & Weitzman, 2014; Kamis, 2021; Scarlett et al., 2024). The transactional way in which parents and children influence each other can contribute to stress in a cyclical way (Biswas et al., 2015; Hayes & Watson, 2013; Mackler et al., 2015; Solem et al., 2011; Whitlock et al., 2018). When symptoms are present outside the home, such as challenges at school, they continue to impact a parent’s level of distress (Anthony et al., 2005). Children with additional care needs or disabilities (e.g., autism) are also more likely to experience problematic family functioning, which includes higher levels of parent stress and marital conflict (Desquenne Godfrey et al., 2024; Hayes & Watson, 2013; Priego-Ojeda & Rusu, 2023). Parental distress is often heightened due to child-related issues, including internalizing and externalizing problems, executive functioning difficulties, and poor child problem-solving (Hutchison et al., 2016; Polizzi et al., 2021; Qian et al., 2024; Seipp et al., 2024). Furthermore, it has been found that children’s executive functioning mediates the relationship between parenting stress and parenting quality (Qian et al., 2024).

Negative life events experienced by parents may further impact the quality of their parenting (Heinrich, 2014; Koerber et al., 2023; Wang & Li, 2023). Major stressors which result in changes to the family structure (e.g., loss of a home, extreme illness) are potentially impactful to family wellbeing and may exacerbate parenting challenges. These events, such as divorce, economic hardship, or natural disaster, impact family functioning and parenting abilities, resulting in reduced warmth, sensitivity, and responsiveness while increasing harsh disciplinary practices (Masarik & Conger, 2017; McDermott & Cobham, 2012; Sarmadi & Khodabakhshi-Koolaee, 2023). These stressors can also lead to parental mental health issues, requiring the need for community resources and supports, including preventative intervention (Seipp et al., 2024; Sherman & Hooker, 2018).

### 1.3. Parental Supports

To counteract distress and promote family functioning, having access to social supports has been shown to positively impact family and parent wellbeing (Fang et al., 2024; Gouin et al., 2016). Research shows that informal (e.g., unpaid respite) and formal support (e.g., engagement with systems) provide similar benefits to parent and family wellbeing, with informal supports being a better predictor of these benefits across multiple studies (Dunst, 2023). For instance, perceived social support is related to positive parenting behaviours (e.g., sensitivity, responsiveness) and wellbeing for families navigating a child’s mental health challenges (Dunst, 2023; Whitlock et al., 2018).

### 1.4. Quality Indicators

Addressing parental needs through reducing distress, promoting parenting strengths, and needs-based interventions may help promote overall familial functioning through the transactional way in which children’s wellbeing impact parents (Sameroff, 2009; Swartz & Collins, 2019). Understanding the quality of services aimed at achieving such goals is important in ensuring the continued provision of impactful intervention. However, quality assessment and monitoring of children’s mental healthcare is currently a challenge. For example, within Canada, the lack of standardized assessment tools prevents accurate comparison and measuring service outcomes for benchmarking (Duncan et al., 2018; Yang et al., 2016). Lack of standardized metrics to monitor the quality of care leads to inconsistent comparisons across agencies (Bailey et al., 2022; Ramalho et al., 2019). The lack of understanding pertaining to children’s mental healthcare is highlighted in a recent Auditor General of Ontario’s report (Auditor General of Ontario, 2025). The report outlines the lack of quality assurance and data tracking for services provided, specifically with regard to client outcomes (Auditor General of Ontario, 2025). A potential solution to this lack of understanding is using an already widely implemented assessment tool to assess service outcomes in the province of Ontario. Many mental health facilities in Ontario utilize the interRAI Child and Youth Mental Health Assessment (ChYMH) as a part of their standard of care (Stewart et al., 2015). As such, the ChYMH offers a potential solution through the development of Quality Indicators (QIs).

In physical healthcare systems, the development of QIs to ensure high-quality, equitable services is a common practice (Deckert et al., 2024; Ettorchi-Tardy et al., 2012; Nerenz et al., 2021; Samartzis & Talias, 2019). QIs calculate a proportion of clients meeting certain criteria (e.g., meeting a desired outcome) at different agencies or in different regions. The agencies each produce a QI result (e.g., 90% of clients met the desired outcome) for the selected criteria. Calculation of QIs occurs at regular intervals (e.g., every year, every quarter). A set of reliable family wellbeing QIs based on standardized, homogenous data would allow children’s mental health services to track outcomes and set benchmarks for quality of care (Ettorchi-Tardy et al., 2012; Guthrie et al., 2022). QI development also incorporates risk adjustment, a process which mathematically accounts for the heterogeneity of cases between providers to allow for fair quality of care comparisons regardless of underlying clientele differences (Houseworth et al., 2022; Lane-Fall & Neuman, 2013; Nerenz et al., 2021). Risk adjustment allows for an accurate assessment of performance, quality of care, and outcomes, preventing unfair penalties or rewards based on the health status of clients (Houseworth et al., 2022). Furthermore, it aids in fair policy evaluation and reduces bias, ultimately contributing to reliable results (Lane-Fall & Neuman, 2013).

### 1.5. Purpose of Study

The complex, transactional relationship between parent and child wellbeing necessitates the understanding of family-related outcomes following involvement with children’s mental health services. Given that there is an existing valid and reliable assessment system in use for most agencies across Ontario, there is an opportunity for the development of QIs for family and parent-related outcomes following involvement with community mental health services. The purpose of this study was to develop a set of risk-adjusted outcome QIs related to caregiver distress, parenting strengths, and family functioning using a standardized comprehensive children’s mental health assessment instrument in service agencies across Ontario, Canada. Given the interplay between children’s mental health and the family system, these indicators will be valuable in better understanding the landscape of children’s mental health services.

## 2. Materials and Methods

### 2.1. QI Calculation and Risk Adjustment Process

Outcomes related to caregiver distress, family function, and parenting strengths were identified in the interRAI ChYMH and ChYMH-DD assessment instruments (Stewart et al., 2015). Outcome QIs are calculated as a proportion, or percentage, of clients meeting predetermined criteria (see methodology) at eligible agencies. Supporting these rates is a process of risk adjustment that seeks to make values more comparable across agencies or over time, since the underlying risk of the outcome can vary based on the presenting characteristics of cases at programme entry. The methodology was based on the Canadian Institute for Health Information’s (CIHI) process for adjusting home care QIs (Canadian Institute for Health Information, 2013). Two primary processes as outlined by CIHI constitute the risk adjustment procedures used in this study: direct adjustment and indirect adjustment. In direct adjustment, the reported population is assigned to three strata of risk based on baseline status, and then performance by an agency among cases in each stratum is weighted based on a standard population to obtain that agency’s rate (Canadian Institute for Health Information, 2013; Jones et al., 2010). This is similar to how age direct adjustment might be applied to cancer rates as population age distribution changes over time. Prior to the weighted additions of the strata, indirect adjustment using other covariates related to the outcome, but not the agencies’ care, is applied to adjust the rates within the strata using logistic regression.

### 2.2. Sample

Data were acquired via the use of two standardized assessment instruments, the interRAI ChYMH and ChYMH-DD. A number of child and youth mental health service agencies in Ontario have voluntarily used these instruments as part of usual clinical practice since 2015. These agencies vary in their clinical specialization, size, and residential treatment capacity. The ChYMH and ChYMH-DD form a part of the Child and Youth suite of interRAI assessment tools, in which the scales and algorithms demonstrate robust psychometric properties (see Stewart et al., 2022 for more information). The ChYMH-DD is substantially similar to the ChYMH but is developed for use with children and youth with developmental disabilities and, therefore, additional items are added specific to this clinical population and other items not applicable to this population are dropped. Each instrument records over 400 clinical items and supports calculation of scales and algorithms that help clinicians in obtaining a data-driven picture of the child/youth’s strengths, needs, functioning, and areas of risk. Assessors in these agencies receive training on the completion of these assessments and include psychologists, nurses, psychiatrists, speech and language therapists, child and youth workers, developmental social service workers, and social workers. All available sources of information (i.e., family members, community members, document review, and clinical observations) are utilized to complete these assessments. Secure web-based software was implemented to record assessment information, requiring responses of the proper form for all essential items before the record could be authorized as complete.

Before making the data available for analysis, personal identifiers were removed. Data sharing in this manner is part of the licence agreement for the use of these instruments. Data from assessments completed between 1 April 2016 and 31 March 2024 were used, selected to produce consecutive assessment pairs that were between 30 and 365 days apart. The fiscal year of the second assessment defines the reporting period, and if an individual had more than one pair for a fiscal year, the last one was selected. This study was approved by the University of Western Ontario’s ethics board (REB#: 124759).

### 2.3. Outcome/Dependant Variable

Caregiver distress was operationalized using two items: parent/primary caregiver is unable or unwilling to continue in caring activities, and parent/primary caregiver expresses feelings of distress, anger, or depression. These two items have been used previously for this purpose (Mitchell et al., 2015). The term “caregiver” for this QI was used to be inclusive of all those caring for children and to maintain consistency with Mitchell et al. (2015). Each item is scored either no, yes, or not applicable (cases where there was no parent or primary caregiver were excluded). If either one or both items are scored as yes, then caregiver distress is deemed to be present.

Family functioning uses four items: a strong and supportive relationship with the family is absent; family are persistently hostile or critical towards the child; family members report feeling overwhelmed by the child; and the parent/primary caregiver is unable or unwilling to continue in caring activities. The first item is scored no or yes, while the last three items can be no, yes, or not applicable. As in caregiver distress, if any assessment score for these last 3 items was not applicable, the case was excluded. If an item was scored yes, it contributed one to a family functioning sum. Higher sums mean less desirable family functioning. Two additional items that were used in a broader family functioning scale were examined for inclusion but subsequently not used: parent/primary caregiver has current developmental or mental health issues, and sibling has current developmental or mental health issues. Compared to the 4 items utilized, these were viewed as less likely to be influenced by effective clinical practice and in fact were distinctly less likely to change over time than the other items.

There were six items that applied to parenting strengths: communicates effectively with the child/youth; assists the child/youth with the regulation of emotions; uses appropriate disciplinary practices; demonstrates warmth and support; shows appropriate supervision and monitoring; and shows appropriate limit setting or expectations. Each of these items is scored by the assessor as either most of the time, occasionally, rarely or never, or no parent/primary caregiver. As in the other domains, if any of the six items was scored as not having a parent or primary caregiver, the case was excluded. Each item was then dichotomized based on the “most of the time” response counting as one, and the six items summed. Higher sums mean less desirable parenting strengths.

For each of these domains, two forms of a QI could be calculated using the baseline and follow-up assessments in each pair: improvement (where the follow-up state was more desirable than the baseline) and worsening (where the follow-up state was less desirable than the baseline). Pairs for which change was not detectable due to baseline states being at minimum or maximum values (i.e., no improvement or worsening was detectable) were excluded from that particular indicator’s analysis and calculation.

### 2.4. Stratification for Direct Adjustment

The Resource Intensity for Children and Youth version 2 (RIChY-2) scale was developed on case mix principals for the ChYMH and ChYMH-DD as a 10-point scale, with higher values representing cases likely to be using more types of services due to their greater clinical needs and complexity (Stewart et al., n.d.). Cut points were made along the RIChY-2 distribution for each subset of cases that could contribute to the QI (i.e., it was possible to improve for an improved version of the QI) to create low, middle, and high strata, with proportions roughly at 20%, 60%, and 20%, respectively.

### 2.5. Covariate Risk Adjustors

Covariate adjustors are taken from the baseline assessment and were identified based on their availability in the ChYMH and ChYMH-DD instruments for which association with clinical change in these domains was thought to be likely. We sought to include measures that described clinical aspects of the child/youth and measures related to the family, as well as other adjustors such as inpatient status or time between assessments. With a wide range of measures available, an exhaustive approach across all QI variations was impractical. Measures were selected based on known clinical association and observed correlations with the outcomes. We applied candidate covariates in backwards elimination multivariate logistic regression models where the QI outcome was the dependent variable, retaining items where *p* < 0.1.

A candidate set of 14 measures were identified as potential covariates:

1. Related to the child or youth (8 measures): sex, age, any of 4 traumas (sexually abused or assaulted, verbally abused or assaulted, emotionally abused, or having witnessed domestic violence), consideration of self-injury, being disruptive or showing poor productivity at school, high externalizing behaviours (based on stealing, elopement, bullying peers, preoccupation with violence, violence to others, intimidation of others, violent ideation, impulsivity, physically abusive behaviour, outbursts of anger, defiant behaviour, and argumentativeness), worsening of psychiatric symptoms, and inadequate problem solving;
2. Related to parents or the family (4 measures): unavailable unpaid support (based on five domains of need: crisis situations, financial problems, babysitting, emotional support, and respite), a parent has developmental or mental health issues, a parent or primary caregiver has experienced a major life stressor, and caregiver anger, depression, or distress (not applied to the caregiver distress QIs);
3. Related to the provider or system (2 measures): inpatient status, and 6 or more months between the baseline and follow-up assessments.

## 3. Results

There were 14,892 contributing pairs of assessments. Table 1 lists the candidate risk adjustors and indicates which ones were retained, based on their significance in a multiple logistic regression model with the QI outcome. All six of the indicators used one or more adjusting measures from each of the three broad areas. Also shown are the RIChY-2 scale values that determine the strata membership based on the roughly 20%/60%/20% distribution of the sample that is eligible for that QI’s calculation.

**Figure 1:**
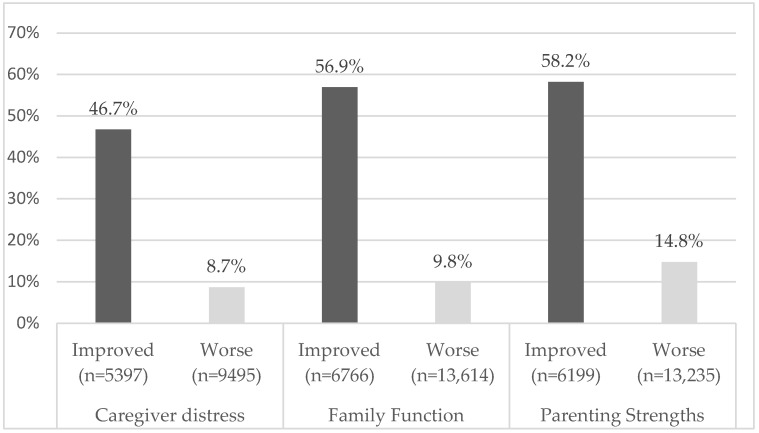
Quality Indicator unadjusted overall rates (with numbers (n)).

Figure 1 shows the overall unadjusted QI rates from the sample dataset, including the number of contributing pairs eligible for each QI. The effective sample size for the improvement version of each QI domain was lower than for worsening, since fewer cases had the issue for which improvement could be detected. Rates of improvement among those with the issue were always much higher than rates of worsening for each of the three domains. Table 2 presents the sample proportions for the risk adjustment covariates and is useful for understanding the sample characteristics.

**Figure A1:**
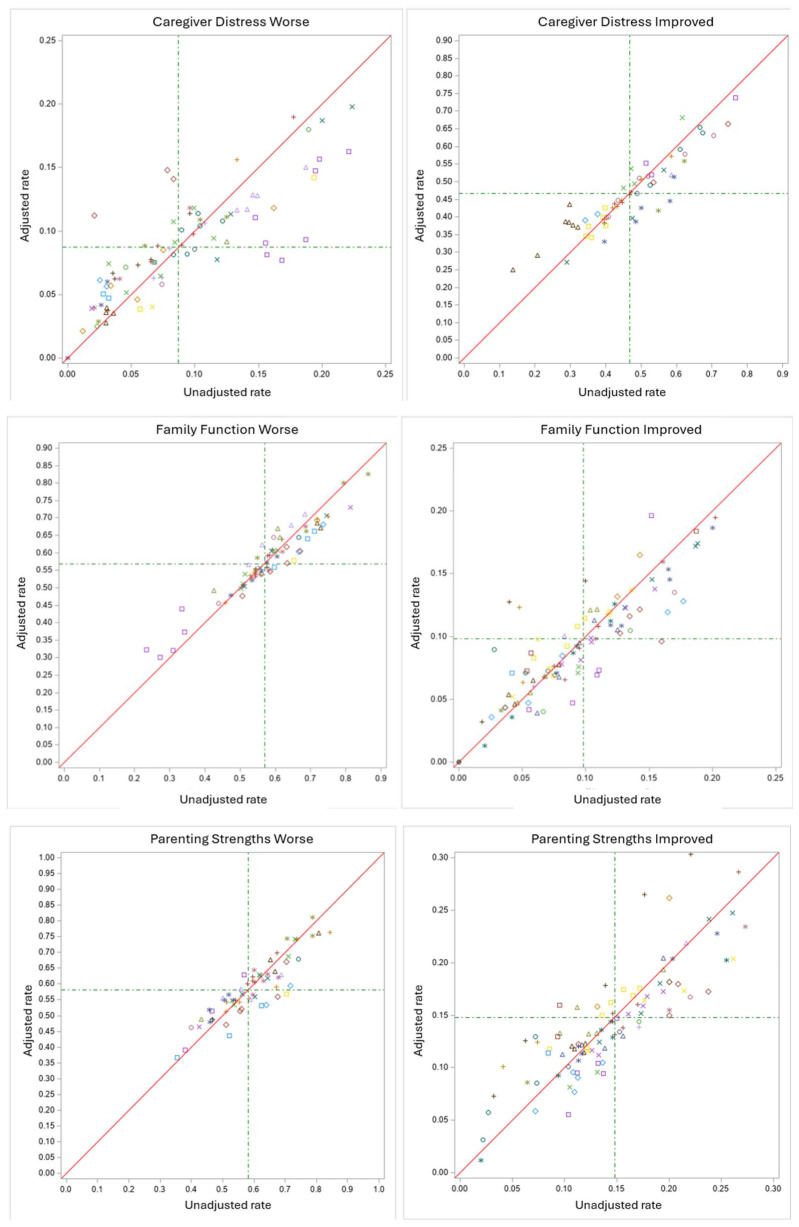
X-Y plots of agency raw and adjusted rates. Different colours reflect specific agencies.

Table 3 summarizes key performance metrics of each of the six QIs. An agency for a given year required a minimum of 30 total assessment pairs to be eligible to compute an adjusted rate, and this resulted in varying numbers of rates depending on the QI: from 58 to 96. The worsening version of each QI resulted in higher numbers of rates, as expected from the higher number of contributing assessment pairs. Distributions of adjusted agency rates shows good variation, as seen in Figure A1, where raw and adjusted rates are plotted. Key correlations in Table 3 are the raw and adjusted values, which range from 0.85 to 0.96, and the corresponding values are provided where stratification alone is used for adjustment, without the covariates. This shows that both the stratification and the within-strata regression using covariates contribute to the adjustment for each of the 6 QIs.

Table 4 presents a correlation matrix of the adjusted QI rates at the agency level, to show how these 6 signals of service quality relate to one another. Consistent modest positive correlations are seen among the 3 improvement QIs (0.63, 0.38, and 0.42), as well as among the 3 worsening QIs (0.46, 0.46, and 0.54), suggesting that improvement or worsening in one domain is associated with an improvement or worsening in the other two domains. Within each of the 3 domains, correlations of improvement and worsening versions (shaded diagonal of the upper right quadrant) are negatively correlated, around −0.4 for caregiver distress and parenting strengths and −0.16 for family function; this indicates that higher rates of improvement tend to be related to lower rates of worsening for a given domain measure, with family function being the least pronounced. Finally, considering correlations for different domains where one is an improvement and the other worsening, we see low correlations, except for family function improvement negatively correlating with caregiver distress worsening (rho = −0.39). Taken together, while most of the correlations are statistically significant, they are mostly modest in magnitude, suggesting that all six of these measures contribute with some degree of independence to the quality profile of an agency.

## 4. Discussion

This study sought to develop a set of risk-adjusted outcome QIs to assess the improvement or decline of family functioning, parenting strengths, and caregiver distress in children receiving mental health support from service agencies in Ontario, Canada. Our study resulted in the calculation of six key QIs, along with a risk adjustment approach to provide, fair, equitable comparisons across time and across agencies. Using existing data, it was demonstrated that improvement in caregiver distress, family functioning, and parenting was observed in approximately half of assessment pairs following service use. In contrast, worsening of these domains was seen in between 8 and 15% of assessment pairs. Our results also show that among the three domains, there are modest relationships between improving (or declining) in one area and improving (or declining) in another area. The modest relationship between interrelated QIs therefore suggest that each of these specific domains are important to independently assess when ascertaining the quality of mental health services.

### 4.1. Notable Child/Youth Level Covariates

To improve the effectiveness of the QIs, various covariates were utilized for risk adjustment. Upon examination of specific child demographics, several influential variables emerged. Parenting (e.g., improved communication, parental warmth, appropriate disciplinary practices) was more likely to improve and less likely to worsen following service engagement if the child was male. This result could be related to the fact that many parenting programmes focus on addressing disruptive, non-compliant behavioural issues, rather than anxiety-related issues (Forehand et al., 2013), and the former often present more often in boys than girls. It may also be that males exhibit more overt presentations (e.g., defiance, non-compliance) which can be addressed more quickly through parenting interventions than the more covert issues that are often expressed by girls (e.g., anxiety, depression).

Caregiver distress (e.g., a parent being overwhelmed, depressed, or angry), family functioning (e.g., a supportive environment), and parenting were less likely to improve and more likely to worsen if *high* levels of externalizing symptoms (regardless of gender) were present. High levels of externalizing symptoms have negative impacts on overall family wellbeing (Fang et al., 2024; Mackler et al., 2015; Solem et al., 2011). For example, serious externalizing difficulties have been demonstrated to overwhelm parents, leading to experiences of distress, shame, and a reduction in engagement with the community (Ringer et al., 2020; Solem et al., 2011). Caregiver distress was also more likely to worsen at follow-up if there was a recent increase in child psychiatric symptoms overall (e.g., depression, anxiety, conduct problems). Consistent with previous research, pediatric help-seeking is often related to high levels of caregiver distress due to acute psychiatric distress (Bowden et al., 2022). Indeed, caregiver strain has been associated with the severity and acuity of the child’s illness, coupled with their social support and other social determinants of health (Mendenhall & Mount, 2011).

With respect to symptom duration and caregiver distress, older age of a client predicted higher rates of caregiver distress with moderate resource needs but lower caregiver distress in those with high service needs. Access to additional resources (e.g., inpatient services, respite, or therapy) may be more readily available for families who have been receiving care for some time, especially those children with greater case complexity (Gallant & Good, 2023). It is also possible that as the length of time for which a parent navigates complex child needs increases, the stress faced by parents may become normalized, resulting in lower distress (Knafl et al., 2010). Consequently, parents may adapt and reorganize their life circumstances to cope (Gray, 2006). Parental resiliency may also play a significant role as parents learn new strategies to manage their level of distress (F.-Y. Lin et al., 2013), while also experiencing greater formal supports and resources when a child’s needs are more complex (Goudie et al., 2014; Government of Ontario, 2019; Greenspan et al., 2015; Ono et al., 2019; Penner et al., 2018).

Childhood trauma had a profound impact as a risk adjuster for all three QI domains, increasing the likelihood of worsening while also reducing the likelihood of improvement for both parenting and family functioning. Consistent with previous research, childhood trauma has a detrimental impact on treatment response, disrupting coping strategies following adversity throughout development (Bahmani et al., 2023; Downey & Crummy, 2022). Children who experience polyvictimization are at increased risk for attachment difficulties, lack informal support, are at higher risk for interpersonal conflict, and are at increased risk for higher levels of aggression and violence (Stewart et al., 2021a). Research has also demonstrated an increase in parental overprotection and avoidance following acute trauma, thereby impacting key parenting practices such as emotional distancing and avoidance, poor communication, and the inability to set limits with their children (Afzal et al., 2023; Bahmani et al., 2023; Steele et al., 2016).

Findings indicated that children who had considered self-injury in the last year were less likely to experience improvements with respect to family functioning. The extant literature has indicated that family dysfunction is often associated with suicidal ideation and attempts in children (Li et al., 2025). Specifically, parents who exhibit emotionless control, insecure attachments, and negligent parenting have children who are more likely to exhibit suicidal ideation and intent (Alvarez-Subiela et al., 2022). Moreover, parents of children engaging in self-harm report feelings of helplessness, self-blame, stress, anxiety, and guilt related to their child’s behaviours (Ferrey et al., 2016; Raphael et al., 2006; Whitlock et al., 2018). Child self-injury is also associated with strained relationships within and outside the family system, including poor communication, lack of adaptability, and high levels of family conflict (Gao et al., 2024; Kelada et al., 2016; Lipschitz et al., 2012; Ren et al., 2018). As such, our results demonstrate that the consideration of self-harm negatively impacts improvements in family functioning during service engagement.

Disruptiveness and poor productivity in school impacted all three QI domains. Essentially, family functioning was less likely to improve and more likely to worsen if the child was disruptive and unproductive in school. Moreover, caregiver distress and parenting worsened if the child was experiencing these school challenges. This is consistent with the previous literature (e.g., Anthony et al., 2005). Additionally, research has demonstrated that family dysfunction and problematic parenting impact school achievement and behaviour, suggesting a transactional process among the child, parent, and contextual factors (Fernández-Alonso et al., 2017; Gana et al., 2024; González Moreno & Molero Jurado, 2022; Kroneman et al., 2009; Mesman et al., 2009; Rayan et al., 2022; Sadiq Sangawi et al., 2015).

Family functioning was less likely to improve and more likely to worsen if the child exhibited inadequate problem solving and reasoning ability. This is consistent with previous research indicating that family functioning, rather than family structure, impacts informal reasoning ability and problem solving, as well as specific academic skills (Chng et al., 2014; Creswell et al., 2017; Friedberg & McClure, 2015; Y.-C. Lin et al., 2019). It was also noted that parenting was more likely to worsen if the child exhibited difficulties in solving everyday problems and making inferences. Specifically, research has indicated that parenting styles are highly associated with problem solving skills (X. Lin et al., 2023), where those children who had more flexible, effective problem-solving skills were more likely to have involved parents who exhibited flexible authoritative, rather than authoritarian, parenting styles. As such, parenting styles have been highly associated with academic skill development, social skills, and cognitive abilities (Rinaldi & Howe, 2012; Roopnarine et al., 2006; Rudasill et al., 2013).

### 4.2. Notable Parent/System-Level Covariates

Social support plays a crucial role in mitigating caregiver distress, poor parenting, and dysfunction within the family (e.g., Dunst, 2023; Fang et al., 2024; Gouin et al., 2016; Whitlock et al., 2018). Consistent with such previous research, caregiver distress, parenting, and family functioning were less likely to improve if there was a lack of informal/unpaid supports. Further, without these supports, caregiver distress and parenting were more likely to worsen. Parent mental health/developmental concerns were related to a decreased likelihood of caregiver distress improvement and an increased likelihood of caregiver distress worsening (Fang et al., 2024). Parents with a developmental disability also face significant challenges compared to parents without, including additional socioeconomic disadvantages and mental health concerns, which could contribute to caregiver distress (Feldman & Aunos, 2020; Heifetz et al., 2019). Parents with developmental and intellectual disabilities also reported feeling judged by, and facing conflict with, different systems (e.g., education; Heifetz et al., 2019). Caregiver anger, depression or distress was related to a decreased likelihood of improving in family functioning and parenting, and an increased likelihood of declining in family functioning and parenting. These results align with previous research demonstrating that parent mental illness contributes to inappropriate parenting practices and challenges in maintaining good relationships with family members, including children (Fang et al., 2024). Lastly, a parent experiencing a recent major stressor (e.g., losing their home) led to higher likelihood of worsening in all three QI domains and a decreased likelihood of caregiver distress improving, consistent with research demonstrating the impact of major stressors on family wellbeing (Koerber et al., 2023; Sarmadi & Khodabakhshi-Koolaee, 2023). Furthermore, it is important to note that inpatient status detrimentally impacted improvement and increased the likelihood of worsening across multiple QI domains.

### 4.3. Limitations

While this study has several strengths (e.g., large data base, comprehensive assessment), one of the major limitations to this work is the lack of standardization across agencies and lack of follow up assessments available. For instance, this study included a fair number of agencies (over 25) in its calculations, yet only agencies which use the ChYMH assessment can be included in this work, and as such agencies in Ontario that do not use this system are inherently excluded. Recently, there have been ongoing calls to mandate such an instrument. Moving to a uniform reporting style will help facilitate the calculation and implementation of benchmarking and quality analysis through outcome QIs. Lack of uniformity in data and reporting has been repeatedly cited as a barrier to obtaining an understanding of the quality of children’s mental health services (Duncan et al., 2018; Junek, 2012). To implement benchmarking, agencies must continue to implement standardized follow-up testing to ensure the maximum number of clients are included in data analysis. This is important as larger data pools allow for more accurate QI calculations (Berg et al., 2002).

A further limitation of this study is that the data used was limited to Ontario only. Potential differences between regions, for instance, differing parenting policies (e.g., parental leave allotment; Heshmati et al., 2023) and cross-cultural differences in parenting practices (e.g., parental discipline; Lansford, 2022), may impact the applicability to family outcomes in other countries. Future work to expand this research to other nations and continents to conduct comparisons is needed and is currently underway. This is an important consideration, given the international uptake of interRAI assessments in various countries (see the development of multinational interRAI homecare QIs as an example; Morris et al., 2013). Further, interRAI self-report assessment instruments are now being piloted internationally (e.g., India, Sri Lanka, Egypt), including in some of the United Nations’s (UN, 2024) least developed nations (e.g., Rwanda, Uganda) utilizing the same applications. Given that interRAI instruments are implemented across over 40 nations, their use provides unique opportunities to make global comparisons.

### 4.4. Future Opportunities for Quality Assessment

Given the accessibility of the interRAI tools due to their widespread implementation and the risk adjustment process developed, a key outcome of this study is the possibility of developing and implementing benchmarking standards of care in children’s mental healthcare. As described, benchmarking allows for the continued monitoring of service indicators to maintain a base level of quality across agencies (Ettorchi-Tardy et al., 2012). Agencies should have the ability to understand how their practices compare to other agencies; stakeholders should have the ability to set standards of care to ensure quality. Our comprehensive family-level outcome QIs contrast with the current quality tracking for family outcomes by the province outlined in the Auditor General’s Report, which at this time rely on satisfaction with services rather than evidence-based outcome measurement. As outlined by Donabedian (Donabedian, 1983, 1985, 2005), outcome measurement is one of the most important types of quality analysis to ensure services are performing at adequate levels from a macro perspective. Given how inseparable the wellbeing of families and children is, having the ability to track family outcomes will ensure stakeholders can understand the larger picture of children’s wellbeing following care.

QIs can have favourable impacts on systemic levels of change, for instance, in provincial policy and resource allocation for agencies. The Auditor General of Ontario (2025) recommends implementing outcome targets (benchmarks) for agencies to meet; mandated reporting of QIs through a uniform reporting measure, such as the interRAI Child and Youth suite of instruments, could facilitate this. There is further potential to incentivise regular QI reporting through the linking of QI performance to agency funding and to enhance the accountability of agencies through the public reporting of these performance benchmarks (Duncan et al., 2018). Once reporting is standardized, resources can be more accurately directed towards agencies whose benchmarks and targets are not being met or to agencies that are identified as struggling with certain outcome measures. Further, the Auditor General of Ontario’s (2025) report on children’s mental healthcare recommends that outcome indicators be used longitudinally (e.g., at an intra-agency level) to identify possible reductions in performance over time. Information on such indicators of performance can be presented to various stakeholders (e.g., through report cards) and has the potential to support arguments for increasing mental health resources through data-informed evidence (Hermann et al., 2007). QI tracking and comparison could also identify high-performing agencies. These agencies can then be consulted to understand which practices are being used to improve care across regions.

It should be noted that this current work is produced in the context of a larger QI development initiative from interRAI. QIs related to client outcomes (e.g., internalizing/externalizing symptoms, self-harm) are concurrently under development; hence, these family-level QIs are only one piece of the puzzle when understanding the quality of mental healthcare for children and youth.

## 5. Conclusions

We have demonstrated the valid development of QIs related to the wellbeing of caregivers and families of children involved with community mental healthcare in Ontario. These QIs, although conceptually related, demonstrated correlations indicative of the measurement of independent constructs, each important understanding the outcomes of children’s mental healthcare. The development of these, and other, outcome measures for children’s mental healthcare in Ontario is key in understanding of the performance of publicly funded children’s mental healthcare services. Ideal outcomes and next steps informed by the results of this study, such as benchmarking, are possible only when assessment tools are available, accurate, and uniform across providers.

## Figures and Tables

**Table 1:** Risk adjustment measure summary.

		Caregiver Distress		Family Function		Parenting	
		Improved	Worse	Improved	Worse	Improved	Worse
Child/ youth	Male					↑	↓
	Greater age	↓	↕	↓	↑	↓	↑
	Any of 4 traumas		↑	↓	↑	↓	↑
	Considered self-injury in last year			↓			
	Disruptive/poor productivity at school		↑	↓	↑		↑
	High level of externalizing behaviours	↓	↑	↓	↑	↓	↑
	Psych. symptoms worse in last 30 days		↑				
	Inadequate problem solving			↓	↑		↑
Parents/ family	Unavailable unpaid support	↓	↑	↓		↓	↑
	Parent has developmental/MH issues	↓	↑				
	Major life stressor in last 90 days	↓	↑		↑		↑
	Caregiver expresses anger, depression, or distress			↓	↑	↓	↑
Provider/ system	Inpatient	↓	↑	↓	↑		↑
	6+ months between assessments	↑	↑	↑	↑	↑	↑
RIChY-2 scale values	Assigned to low strata	1–5	1, 2	1–5	1–3	1–4	1–3
	Assigned to middle strata	6–9	3–7	6–9	4–8	5–8	4–8
	Assigned to high strata	10	8–10	10	9, 10	9, 10	9, 10

*Note.* Entries indicate where a covariate was significant at *p* < 0.1 in a multivariate logistic regression model in one or more strata out of the three (↑ = predicts higher rate of the outcome; ↓ = predicts lower rate of the outcome; ↕ = inconsistent association in the three strata).

**Table 2:** Characteristics of the overall sample at the baseline assessment, including risk-adjusting covariates.

	Prevalence (All)
N (assessment pairs)	14,892
Quality Indicator outcomes:	
Caregiver distress improved (of 5397 eligible pairs)	46.7%
Caregiver distress worsened (of 9495 eligible pairs)	8.7%
Family function improvement (of 6766 eligible pairs)	56.9%
Family function worsened (of 13,614 eligible pairs)	9.8%
Parenting strength improvement (of 6199 eligible pairs)	58.2%
Parenting strengths worsened (of 13,235 eligible pairs)	14.8%
Covariate prevalence	
Sex: male	52.7%
Age: 4 to 7	13.2%
8 to 11	33.9%
12 to 14	27.7%
15 to 18	25.2%
Mean (std)	11.7 (3.4)
Any of 4 traumas	41.9%
Considered self-injury in last year	34.8%
Disruptive/poor productivity at school	33.3%
High level of externalizing behaviours	19.6%
Psychiatric symptoms worse in last 30 days	12.1%
Inadequate problem solving	44.2%
Unavailable unpaid support	23.5%
Parent has developmental/MH issues	46.2%
Major life stressor in last 90 days	25.6%
Caregiver expresses anger, depression, or distress	35.2%
Inpatient	6.2%
6+ months between assessments	29.7%

**Table 3:** QI adjustment summary.

		Adjusted Agency Rate Percentiles					Correlation: Raw and Adjusted Rates	
Quality Indicator	Number of Rates	P5	P20	Median	P80	P95	With Covariates	Strata Alone, No Covariates
Caregiver distress improved	58	26.9%	39.2%	45.1%	54.5%	64.4%	0.927	0.979
Caregiver distress worse	86	2.8%	5.5%	8.5%	12.7%	16.6%	0.850	0.942
Family function improved	69	37.3%	51.2%	57.8%	66.9%	71.1%	0.957	0.984
Family function worse	96	3.2%	6.0%	9.1%	12.3%	17.4%	0.874	0.917
Parenting strengths improved	62	44.3%	50.4%	57.7%	65.6%	79.4%	0.904	0.966
Parenting strengths worse	96	6.0%	10.8%	13.4%	17.5%	25.5%	0.866	0.942

**Table 4:** QI agency rate correlation matrix.

Pearson Correlation of Agency Rates; n Varies from 51 to 94		Quality Indicator: Improvement			Quality Indicator: Worse		
		Caregiver Distress	Family Function	Parenting Strengths	Caregiver Distress	Family Function	Parenting Strengths
Quality Indicator: improvement	Caregiver distress		**0.63**	**0.38**	**−0.38**	−0.06	−0.08
	Family function			**0.42**	**−0.39**	−0.16	0.05
	Parenting strengths				−0.15	−0.24	**−0.44**
Quality Indicator: worse	Caregiver distress					**0.46**	**0.46**
	Family function						**0.54**
	Parenting strengths						

*Note.* Bolded values indicate significance at *p* < 0.05.

## Data Availability

The datasets presented in this article are not readily available due to licencing agreements and privacy and ethical issues.

## Abbreviations

The following abbreviations are used in this manuscript:

ChYMH	Child and Youth Mental Health Assessment
ChYMH-DD	Child and Youth Mental Health Assessment (Developmental Disabilities)
QI	Quality Indicator
PTSD	Post-Traumatic Stress Disorder
